# c-Met inhibitors

**DOI:** 10.1186/1750-9378-8-13

**Published:** 2013-04-08

**Authors:** Anum Mughal, Hafiz Muhammad Aslam, Asfandyar Sheikh, Agha Muhammad Hammad Khan, Shafaq Saleem

**Affiliations:** 1 Dow Medical College, Dow University of Health Sciences, Karachi, Pakistan; 2 Pakistan Research Evolution Students’ Society, Karachi, Pakistan; 3 Sindh Medical College, Dow University of Health Sciences, Karachi, Pakistan

**Keywords:** c-Met, Tyrosine kinase, Cancer

## Abstract

c-Met is a receptor tyrosine kinase that encodes protein such as hepatocyte growth factor receptor (HGFR). Inappropriate activity of c-Met can cause wide variety of carcinomas. c-Met inhibitor are relatively new class of small molecules that inhibit the enzymatic activity of c-Met tyrosine kinase. Met inhibitors divided into two main classes: class I (SU-11274-like) and class II (AM7-like). The use of c-Met inhibitors with other therapeutic agents could be crucial for overcoming potential resistance as well as for improving overall clinical benefit. Met pathway inhibitors might be used in combination with other treatments, including chemo-, radio- or immunotherapy

## Letter to editor

c-Met is a proto-oncogene that encode a protien known as hepatocyte growth factor receptor (HGFR) [1-3]. It is essential for embryonic development, wound healing and organ morphogenesis [4,5]. MET is a membrane receptor. It stimulates cell scattering, invasion, protection from apoptosis and angiogenesis [6]. MET is normally expressed by cells of epithelial origin [4]. Deregulated activity of c-Met can cause a wide variety of different cancers, such as colorectal, thyroid, renal cell, ovary, breast, pancreas, prostate, liver, and melanoma and in gastric carcinoma [7-11].

Hepatocyte growth factor (HGF) is the only known ligand of the MET receptor [4,5,12]. Upon HGF stimulation, MET induces MET kinase catalytic activity which triggers transphosphorylation of the tyrosine Tyr 1234 and Tyr 1235. These two tyrosines engage various signal transducers, thus initiating a whole spectrum of biological activities driven by MET, collectively known as the invasive growth program. Over expression, gene amplification, mutation, a ligand-dependent auto- or paracrine loop or an untimely activation of RTK leads to c-Met dysregulation [4,13]. Cancer development is closely associated with different oncogenic pathways. RAS pathway mediates scattering and proliferation signals which lead to branch morphogenesis [14]. PICK3 pathways activates either directly or through down stream of RAS pathway [15]. Beta catenin pathway participates in transcriptional regulation of numerous genes while NOTCH pathway activates through Delta ligand [16,17]. Activation of these oncogenic pathways (RAS, PI3K, STAT3, beta-catenin), angiogenesis and cells dissociation due to metalloprotease production, which often leads to metastasis, are involved in the development of cancer [18].

c-Met inhibitors are relatively new class of small molecules that inhibit the enzymatic activity of the c-Met tyrosine kinase. Pyrrole-indolinone (PHA-665752) is a prototype selective class of inhibitors that inhibits HGF/SF-induced receptor phosphorylation [4,5,19]. There are basically two classes of c-Met inhibitors, ATP competitive and ATP non-competitive inhibitor. ATP competitive inhibitors are further divided into two classes; class I (SU-11274-like) and class II (AM7-like) on the basis of different types of binding and a third group of non-competitive ATP inhibitor that binds in a different way to the other two [20,21]. Class I inhibitors are selective, U-shaped and attached to the activation loop of c-Met. JNJ-38877605 ( for advanced and refractory solid tumors) and PF-04217903 are class I met inhibitors that underwent phase I clinical trials in 2010 [22]. Class II inhibitors have urea group in either cyclic or acyclic form. Foretinib XL880 is a class II met inhibitor that targets multiple tyrosine kinases, primary targets are MET, VEGFR2 and KDR. It has completed phase-2 clinical trial with indications for head and neck, gastric and renal cell carcinoma [22,23]. Other candidates undergoing trials include Merck’ MK-2461, Bristol Meyers Squib’ BMS-777607, GSK/Exelixis’ GSK/1363089/XL 880 and BMS/Exelixis’ XL-184 [12,22].

The Met pathway is one of the most frequently dysregulated pathways in human cancer [12]. c-Met inhibitors that are currently in clinical trials include cabozantinib and foretinib. Cabozantinib (XL184) was approved by the U.S. FDA in November 2012 for the treatment of medullary thyroid cancer [24]. Patients taking this medication should not ingest grapefruit or grapefruit juice as it may increase the concentration of the drug in the patient's blood [25]. Foretinib is yet an experimental drug candidate for the treatment of cancer [26].

The use of c-Met inhibitors with other therapeutic agents could be crucial for overcoming potential resistance as well as for improving overall clinical benefit. As a key element in the development of any targeted therapy, the biochemical and molecular determination of the precise functions of the Met pathway in the context of other relevant pro-cancer pathways will undoubtedly play a significant role in this effort [12]. More implications are likely to be discovered as new horizons in cancer therapeutics are unveiled [27].

## Competing interest

Authors declare they have no competing interest.

## Authors’ contribution

AM and HMA did manuscript drafting and AS, AMHK and SS did critical review. All have given final approval of the version to be published.
